# Genome-wide DNA methylation profiling of CD8+ T cells shows a distinct epigenetic signature to CD4+ T cells in multiple sclerosis patients

**DOI:** 10.1186/s13148-015-0152-7

**Published:** 2015-11-05

**Authors:** Vicki E. Maltby, Moira C. Graves, Rodney A. Lea, Miles C. Benton, Katherine A. Sanders, Lotti Tajouri, Rodney J. Scott, Jeannette Lechner-Scott

**Affiliations:** Centre for Information Based Medicine, Hunter Medical Research Institute, Newcastle, Australia; School of Biomedical Sciences and Pharmacy, University of Newcastle, Newcastle, Australia; School of Medicine and Public Health, Univeristy of Newcastle, Newcastle, Australia; Insitute of Health and Biomedical Innovation, Queensland University of Technology, Brisbane, Australia; Faculty of Health Sciences and Medicine, Bond University, Gold Coast, Australia; Division of Molecular Medicine, Pathology North, Newcastle, Australia; Department of Neurology, Devision of Medicine, John Hunter Hospital, Newcastle, Australia

**Keywords:** Multiple sclerosis, DNA methylation, CD8+ T cells, HLA-DRB1

## Abstract

**Background:**

Multiple sclerosis (MS) is thought to be a T cell-mediated autoimmune disorder. MS pathogenesis is likely due to a genetic predisposition triggered by a variety of environmental factors. Epigenetics, particularly DNA methylation, provide a logical interface for environmental factors to influence the genome. In this study we aim to identify DNA methylation changes associated with MS in CD8+ T cells in 30 relapsing remitting MS patients and 28 healthy blood donors using Illumina 450K methylation arrays.

**Findings:**

Seventy-nine differentially methylated CpGs were associated with MS. The methylation profile of CD8+ T cells was distinctive from our previously published data on CD4+ T cells in the same cohort. Most notably, there was no major CpG effect at the MS risk gene HLA-DRB1 locus in the CD8+ T cells.

**Conclusion:**

CD8+ T cells and CD4+ T cells have distinct DNA methylation profiles. This case–control study highlights the importance of distinctive cell subtypes when investigating epigenetic changes in MS and other complex diseases.

## Findings

Multiple sclerosis (MS) susceptibility is influenced by a combination of genetic factors and environmental exposures. CD4+ T cells have long been favoured as the most important immune cell subset in the pathogenesis of disease, but there is increasing evidence that CD8+ T cells play a substantial role in central nervous system damage (reviewed in [1]).

Despite several large genome-wide association studies (GWAS), there remains a large proportion of unexplained heritability in terms of MS risk. Epigenetics can influence the genome without changes to the DNA sequence. Environmental exposures such as smoking and vitamin D levels have been demonstrated to modify epigenetic mechanisms, providing a plausible link between environmental factors and disease [2, 3]. One such epigenetic mechanism is DNA methylation, which is the addition of a methyl group to CpG dinucleotides. We, and others, have used genome-wide DNA methylation technologies to assess differentially methylated regions (DMRs) of CD4+ T cells in MS patients compared to healthy controls [4–6]. We found a striking methylation signal located on chromosome 6p21 with a peak signal at HLA-DRB1, which remained after controlling for background SNP effects, as well as 55 non-HLA CpGs that localise to genes previously linked with MS.

In an effort to determine if these previously identified DMRs were specific to CD4+ T cells, we performed a genome-wide methylation study of CD8+ T cells using the same cohort, workflow and data analysis as described in our previous study [5]. Briefly, DNA from total CD8+ T cells was extracted from 30 MS patients and 28 healthy age- and sex-matched controls. The DNA was bisulphite-converted and hybridised to Illumina 450K arrays. Raw fluorescence data were processed using a combination of R/Bioconductor and custom scripts of a total of 442,672 probes representing individual CpG sites that passed quality control (QC) steps. These CpGs were analysed by statistical modelling of methylation levels (*β* values) between MS cases and controls.

**Fig. 1:**
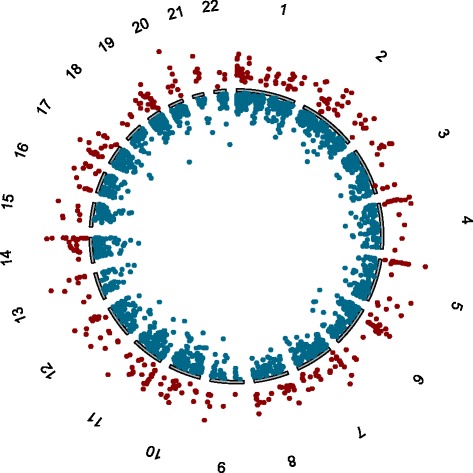
A genome-wide differential methylation plot based on sites passing a nominal *p* value of 0.05*. Data points outside* the circle represent increased methylation in multiple sclerosis (MS) patients compared to controls (i.e. Δ_meth_)_,_ whereas *points inside* the circle represent methylation in the MS group

Figure 1 shows the genome-wide distribution of differential methylation scores for all CpG sites that passed the nominal *p* value cut-off of 0.05. We conducted a stepwise prioritisation strategy to extract the most robust CpG loci associated with MS. Based on the criteria of (i) FDR *p* < 0.05 and (ii) *Δ*_meth_ ≥ ± 0.1 thresholds, 111 CpGs were extracted. To filter out potential effects of gender and treatment, we performed a subgroup analysis of the methylation statistics as previously described [5]. This process reduced the number of associated CpG sites down to a core panel of 79 (Table 1).

**Table 1:** MS-associated CpGs in CD8+ T cells

Probe ID^a^	CHR^b^	Position	Gene^c^	Feature	Median (case)	Median (control)	*Δ*_meth_^d^	*p* value^e^
cg03431738	21	40031295	ERG	5′UTR	0.81	0.68	0.13	0.004033
cg12026095	19	49468461	FTL	TSS200	0.30	0.49	−0.18	0.004033
cg26228123	14	73392919	DCAF4	TSS200	0.09	0.20	−0.11	0.004033
cg10478035	13	80919503		-	0.75	0.64	0.11	0.004033
cg04474988	10	131770171		-	0.34	0.46	−0.11	0.03549
cg25152348	22	50946712	NCAPH2	1st exon	0.30	0.47	−0.17	0.03549
cg08206623	11	2907334	CDKN1C	TSS1500	0.29	0.44	−0.15	0.004033
cg13738615	9	109624741	ZNF462	TSS1500	0.18	0.31	−0.13	0.004033
cg01525244	22	39548611	CBX7	TSS200	0.14	0.24	−0.10	0.004033
cg12702165	12	95228136	MIR492	TSS200	0.65	0.54	0.11	0.004033
cg06443542	10	100206752	HPS1	TSS200	0.14	0.25	−0.11	0.03549
cg00380172	6	148663585	SASH1	TSS200	0.21	0.33	−0.12	0.03549
cg19095187	6	108437051		-	0.17	0.31	−0.14	0.03549
cg04488145	3	46899455	MYL3	3′UTR	0.83	0.73	0.11	0.03549
cg03027241	20	49620453	KCNG1	3′UTR	0.50	0.32	0.18	0.004033
cg11700985	10	82127205	DYDC2	3′UTR	0.85	0.74	0.11	0.03549
cg07886142	5	126793022	MEGF10	3′UTR	0.59	0.46	0.13	0.03549
cg18183163	2	171574141	SP5	3′UTR	0.12	0.26	−0.14	0.03549
cg01181415	12	16757954	LMO3	5′UTR	0.22	0.36	−0.14	0.03549
cg10143811	12	16757985	LMO3	5′UTR	0.12	0.22	−0.10	0.03549
cg23274123	1	229478617	C1orf96	5′UTR	0.10	0.22	−0.12	0.004033
cg00095276	5	1068111	SLC12A7	Body	0.77	0.63	0.15	0.004033
cg03447557	1	2273735	MORN1	Body	0.80	0.70	0.10	0.03549
cg02745847	17	47075880	IGF2BP1	Body	0.17	0.31	−0.13	0.03549
cg09406795	11	64019655	PLCB3	Body	0.25	0.38	−0.13	0.000358
cg18016288	13	95834131	ABCC4	Body	0.47	0.32	0.15	0.000358
cg14486346	2	102000131	CREG2	Body	0.78	0.66	0.12	0.03549
cg21937244	14	103406412	CDC42BPB	Body	0.75	0.61	0.14	0.03549
cg11811840	2	234669166	UGT1A10	Body	0.84	0.72	0.12	0.03549
cg25756617	1	43734917	TMEM125	TSS1500	0.69	0.58	0.11	0.03549
cg03768916	10	49813307	ARHGAP22	TSS200	0.30	0.43	−0.14	0.004033
cg06524757	13	72441523	DACH1	TSS200	0.25	0.35	−0.11	0.03549
cg03168749	11	124413574	OR8B12	TSS200	0.82	0.68	0.14	0.03549
cg21276022	9	136390236	TMEM8C	TSS200	0.74	0.61	0.13	0.004033
cg09851596	8	143545214	BAI1	TSS200	0.60	0.49	0.11	0.03549
cg25296222	11	2037173		-	0.76	0.65	0.11	0.03549
cg00878533	1	2848864		-	0.72	0.62	0.11	0.000358
cg03612700	17	18970610		-	0.64	0.52	0.12	0.004033
cg03310594	7	22704316		-	0.82	0.69	0.13	2.34E-05
cg05854694	14	61123243		-	0.12	0.22	−0.10	0.000358
cg12384499	15	89949617		-	0.19	0.31	−0.11	0.004033
cg22509113	2	91777482		-	0.41	0.51	−0.10	0.004033
cg10495084	15	96889416		-	0.24	0.36	−0.12	0.004033
cg18008019	13	100641646		-	0.10	0.23	−0.12	0.03549
cg12093775	13	112548065		-	0.15	0.26	−0.11	0.000358
cg12787323	10	119494959		-	0.16	0.27	−0.11	0.004033
cg22792862	14	67827087	EIF2S1	1st exon	0.23	0.38	−0.15	0.004033
cg08969532	10	99790438	CRTAC1	1st exon	0.05	0.15	−0.10	0.004033
cg18185028	3	154042079	DHX36	1st exon	0.30	0.41	−0.11	0.000358
cg23059965	19	50655862	C19orf41	3′UTR	0.81	0.70	0.11	0.004033
cg02192678	8	1495185	DLGAP2	5′UTR	0.78	0.68	0.11	0.004033
cg02976009	6	32068226	TNXB	5′UTR	0.71	0.59	0.12	0.03549
cg18073471	4	81119198	PRDM8	5′UTR	0.18	0.29	−0.11	0.03549
cg00945810	7	814391	HEATR2	Body	0.67	0.56	0.11	0.03549
cg04875614	4	2008706	WHSC2	Body	0.80	0.69	0.10	2.34E-05
cg26920627	1	7319248	CAMTA1	Body	0.75	0.63	0.12	0.004033
cg26647242	2	30040525	ALK	Body	0.78	0.67	0.11	0.004033
cg04605816	20	62092443	KCNQ2	Body	0.83	0.71	0.12	0.004033
cg10944063	2	120233706	SCTR	Body	0.58	0.46	0.12	0.004033
cg14595269	7	151216272	RHEB	Body	0.14	0.24	−0.10	2.34E-05
cg23720125	5	177097760	LOC202181	Body	0.85	0.73	0.12	0.004033
cg02047661	3	51976883	RRP9	TSS1500	0.64	0.52	0.11	0.004033
cg07925549	12	52828840	KRT75	TSS1500	0.75	0.63	0.12	0.03549
cg06697094	17	54911185	DGKE	TSS1500	0.16	0.28	−0.12	0.03549
cg18789663	1	242688591	PLD5	TSS1500	0.09	0.20	−0.11	0.03549
cg03468541	14	89029199	ZC3H14	TSS200	0.17	0.30	−0.13	0.004033
cg13526221	8	987389		-	0.79	0.69	0.11	0.004033
cg03313895	4	24803042		-	0.65	0.54	0.10	0.03549
cg19442593	2	26252851		-	0.85	0.74	0.11	0.004033
cg04851089	6	28953923		-	0.39	0.54	−0.15	0.004033
cg24520975	6	31651362		-	0.86	0.75	0.11	0.03549
cg01932076	21	47394659		-	0.18	0.30	−0.12	2.34E-05
cg17555825	5	76924190		-	0.16	0.26	−0.10	0.03549
cg23154781	15	80634195		-	0.81	0.69	0.12	0.004033
cg00792513	6	100066698		-	0.34	0.47	−0.14	0.03549
cg23708569	14	106058450		-	0.63	0.51	0.13	2.34E-05
cg09579989	12	110685438		-	0.81	0.71	0.10	0.03549
cg12077664	12	125145446		-	0.78	0.64	0.14	0.000358
cg24824082	2	133030701		-	0.24	0.35	−0.11	0.000358

Dash indicates intergenic

*UTR* untranslated region, *TSS* transcription start site

^a^Probe ID on 450K chip

^b^Chromosome

^c^Gene annotated to probe

^d^Differential-methylated score

^e^ *p* value for specified probe in CD8+ T cells

Of the 79 CpGs showing differential methylation in MS patients after filtering, all resided outside the MHC locus on chr 6p21. Of these, 27 were intergenic (34 %), have no gene association, or map to genes of unknown function. Of the remaining 52 loci, 26 % are promoter associated, 9 % are in the 5′UTR, 5 % are in the 1st exon, 20 % are in gene bodies and 8 % are in the 3′UTR. Interestingly, none of these CpGs maps to genes that have previously been reported to have a relationship with MS [7, 8]. There was no overlap between these results and our previous results, and, unlike in CD4+ T cells, there was no gene that contained multiple differentially methylated sites. *MORN1* has a single hypermethylated CpG in both CD4+ and CD8+ T cells; however, it was a different site in each study, making it unlikely that this is a significant finding. Our observations are consistent with the recent study by Bos et al., who also identified minimal overlap between the methylation profiles of CD4+ and CD8+ T cells of MS patients [4].

Using GSEA with WebGestalt, our patient cohort did not have prominent pathways in the KEGG Pathway analysis or disease association analysis. The most significant promoter associated with differential methylation was the ferritin light chain (*FTL*) gene. The MS cohort displayed decreased methylation at this CpG locus compared to controls. The gene’s biological function is cation transport. One of the statistically significant genes, *ERG* (ETS-related gene)*,* had a single hypermethylated CpG in the MS cohort compared to controls. *ERG* is a member of the transcription factor family involved in activities such as cell proliferation, differentiation, apoptosis and inflammation. *FTL* is a component of ferritin, and defects in this subunit are associated with other neurodegenerative diseases where mutations result in accumulation of iron in the brain [9]. Relapsing–remitting multiple sclerosis (RRMS) patients have increased iron deposits in their grey matter as compared to healthy controls; thus, misregulation of *FTL* could be important in disease pathology [10, 11]. Mutations in *DCAF4* are associated with leucocyte telomere length, and there is evidence that shortened telomere length in leucocytes is associated with other neurodegenerative diseases, such as Parkinson and Alzheimer’s disease [12–14]. In addition, one study found a shorted telomere length in primary progressive MS patients, but no correlation between RRMS and differing telomere length has been established [15].

Interestingly, we did not see a cluster of differentially methylated CpGs within *HLA-DRB1* as seen in CD4+ T cells [5]. It is well known that the HLA region is notoriously difficult to investigate with many molecular techniques due to increased genetic variation. To minimise the possibility that our observed methylation profile was due to the probes in this region not meeting QC, we used targeted pyrosequencing on available case and control DNA samples. This assay covered seven of the ten differentially methylated CpGs identified in our previous study, but due to high sequence variability, only five of the seven sites returned data. We calculated the median beta values across the five CpG sites using the K–S test. Results showed that the median methylation level in the cases (median = 3.6) and controls (median = 3.6) was not significantly different (*p* = 0.72). This supports a conclusion that this MS-related DMR at *HLA-DRB1* does not exist in CD8+ T cells but is unique to CD4+ T cells.

A recent study by Bos et al. (2015) also found no major effect loci or clusters of differentially methylated CpGs in the CD8+ T cells of MS patients. However, of the top 40 CpG sites, none overlaps with the top 79 sites found in our study. In addition, we found that approximately half the differentially methylated sites were hypermethylated. This is also in contrast to Bos et al., who found nearly 95 % of sites were hypermethylated in CD8+ T cells. Unlike Bos et al., we chose not to filter out probes that are known to contain SNPs. We reasoned that any false positive signals exclusively due to SNP effects would be subsequently identified by genotyping at the key loci. In support of this notion, pyrosequencing of the key *HLA-DRB1* locus did not alter our array-based findings. Additionally, we did not observe a signal at the *HLA-DRB1* locus in CD8+ T cells but did in CD4+ T cells, providing further support that SNPs are not influencing the findings at this locus.

One important consideration of our study is that the patients were being, or had been, treated with various immunomodulatory therapies at the time of recruitment. In particular, eight patients were being treated with fingolimod, which prevents CD4+ lymphocyte egress from lymphoid tissue. As part of our analysis, we stratified our case–control analysis based on treatment groups in an effort to determine whether overall differential methylation signal may be confounded. None of the patient treatment groups shows a distinct methylation signature, including fingolimod (data not shown), which supports the notion that the small number of treated patients in our cohort is not affecting our results. We do note that this does not necessarily mean that fingolimod is not acting on the methylome, but we can conclude that the small number of patients being treated with fingolimod in our study is not confounding the findings. Future studies will benefit from treatment-naïve patients or will be limiting the study to patients on a particular treatment group.

In this study, we identified 79 CpGs showing minor association with MS. None of these hits was observed in the CD4+ T cells from the same cohort, including the major CD4+ DMR at *HLA-DRB1*. All genome-wide DNA methylation studies to date have used relatively small sample sizes. This has resulted in identification of large-effect regions only. Large-scale studies are needed to identify minor-effect DMRs. Future studies should also examine the functional consequences of these changes through transcript analysis. Primarily, the results of this study highlight the need to focus on individual cell types when assessing DNA methylation associated with MS susceptibility.

### Ethics statement

The Hunter New England Health Research Ethics Committee and University of Newcastle Human Ethics committee approved this study (05/04/13.09 and H-505-0607, respectively). MS patients gave written and verbal consent. The Australian Red Cross Blood Service ethics committee approved the use of blood from healthy donors.

## Abbreviations

DMR: differentially methylated region
DNA: deoxyribonucleic acid
FDR: false discovery rate
GSEA: gene set enrichment assay
GWAS: genome-wide association study
MHC: major histocompatibility complex
MS: multiple sclerosis
QC: quality control
SNP: single nucleotide polymorphism
